# Transforming Growth Factor Beta2 Promotes Migration and Inhibits the Proliferation of Gastric Cancer Cells by Regulating the pSmad2/3‐NDRG1 Signaling Pathway

**DOI:** 10.1002/mco2.70148

**Published:** 2025-03-27

**Authors:** Feng‐Jun He, Xiao‐Long Chen, Yun‐Feng Zhu, Hua‐Yang Pang, Ze‐Dong Li, Pan‐Ping Liang, Tao Jin, Zheng‐Wen Chen, Ze‐Hua Chen, Jian‐Kun Hu, Kun Yang

**Affiliations:** ^1^ Department of General Surgery & Laboratory of Gastric Cancer State Key Laboratory of Biotherapy Collaborative Innovation Center of Biotherapy and Cancer Center West China Hospital Sichuan University Chengdu China; ^2^ Gastric Cancer Center West China Hospital，Sichuan University Chengdu China; ^3^ Department of Thyroid and Breast Surgery West China School of Public Health and West China Fourth Hospital Chengdu China; ^4^ West China School of Medicine West China Hospital Sichuan University Chengdu China

**Keywords:** gastric cancer, metastasis, N‐myc downstream‐regulated gene 1, proliferation, transforming growth factor beta2

## Abstract

Transforming growth factor beta2 (TGFβ2) is upregulated in gastric cancer (GC), playing a crucial role in driving its progression. However, the biological effects of TGFβ2 in GC metastasis and proliferation remain not fully understood. Our study reveals that TGFβ2 enhances N‐myc downstream‐regulated gene 1 (NDRG1) protein expression by activating the TGFβR/Smad2/3‐dependent pathway, accelerating GC progression. TGFβ2 knockdown downregulates NDRG1 by inhibiting the TGFβR/Smad2/3 signaling pathway, which in turn inhibits GC cell migration and epithelial–mesenchymal transition (EMT) but stimulates proliferation. Both TGFβ2 upregulation and NDRG1 upregulation enhance GC cell migration in vitro and promote lung metastasis in mouse models. Interfering with NDRG1 reverses TGFβ2‐induced migration, and inhibiting Smad2/3 or TGFβR reverses TGFβ2‐induced NDRG1 upregulation and GC cell migration. Clinical sample analysis shows high TGFβ2 and NDRG1 expression in GC, associated with poor prognosis. Our study reveals that TGFβ2 upregulates NDRG1 via the TGFβR/Smad2/3 pathway, driving GC progression and highlighting the potential role of the TGFβ2NDRG1 axis in GC‐targeted therapies.

## Graphical abstract

The binding of TGFβ2 to its cognate receptor triggers activation of the Smad2/3 signaling cascade. Following phosphorylation, Smad3 undergoes nuclear translocation where it specifically binds to the NDRG1 promoter region, facilitating transcriptional activation of this target gene. This molecular mechanism ultimately upregulates NDRG1 expression and enhances migratory capacity in gastric cancer（GC） cells.

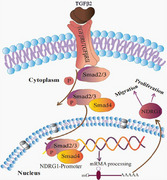

## 1 Introduction

Gastric cancer (GC) ranked as the fifth most prevalent malignancy and the fourth leading cause of cancer deaths globally [1]. Numerous therapeutic advancements have been achieved in the topic of GC during the past two decades, including CAR T‐cell therapy and the disclosing of new molecular targets like CLDN18.2, human epidermal growth factor receptor 2 (Her2), and fibroblast growth factor receptor 2b (FGFR2b) [2, 3, 4]. In clinical practice in China, approximately two‐thirds of GC patients are diagnosed at an advanced stage [2]. Despite advances in the diagnosis and treatment of GC, large proportion of advanced‐stage tumors and tumor metastasis remain the primary causes of death among GC patients [5, 6]. Statistics indicate that metastasis accounts for about 90% of tumor‐related deaths in most cancers [7]. Metastatic tumors often exhibit significant heterogeneity and represent a systemic disease, with most clinically evident metastatic tumors being incurable [8, 9, 10]. The mechanisms of GC metastasis are still not fully understood.

The transforming growth factor beta (TGFβ) family is a group of proteins regulating cell growth and differentiation [11]. Three members have been found in TGFβ superfamily, TGFβ1, TGFβ2, and TGFβ3, which are encoded by distinct genes [11]. They are crucial regulatory factors in embryonic growth and development, participating in and determining the fate of embryonic cell growth and development [11, 12]. Despite the high conservation of amino acid sequences among the mature TGFβ protein subtypes [13], studies have revealed variations among these three subtypes in terms of expression profiles, patterns of functional domain activation, and activation thresholds, resulting in a wide range of biological functions, each with unique specificity [13]. Considering their important oncology roles, TGFβ superfamily has been identified as powerful potential therapeutic target for many years. However, preclinical studies in drug toxicology have revealed that broad‐spectrum inhibition of the TGFβ pathway is associated with toxic side effects such as bleeding, cardiac toxicity, and severe systemic inflammation, thereby significantly impeding the clinical application and promotion of these drugs [13]. Selective blockade can markedly mitigate toxic side effects associated with broad TGFβ signal inhibition [13]. Analyzing public GC databases and clinical samples, we discovered that upregulated TGFβ2 expression in GC is associated with prognosis.

TGFβ2 primarily transmits signals by activating Smad‐dependent and Smad‐independent pathways [14]. The Smad‐dependent pathway involves TGFβ2 binding to TGFβRII, then activating TGFβRI, which phosphorylates Smad proteins. Phosphorylated R‐Smads (Smad2/3) complex with Smad4 enter the nucleus and bind to target gene promoters, regulating transcription [14]. Furthermore, TGFβ2 signals via the phosphatidylinositol 3‐kinase (PI3K)/Akt and mitogen‐activated protein kinase (MAPK) pathways, influencing cell proliferation, migration, and transcriptional regulation [14, 15, 16, 17, 18]. Moreover, TGFβ2 crucially regulates epithelial–mesenchymal transition (EMT), promoting tumor metastasis via transcriptional and posttranscriptional mechanisms [11, 14]. TGFβ2 can play either a protumorigenic or antitumorigenic role at different stages of tumor development, indicating its complex role in cancer biology. However, the molecular mechanisms underlying its role in GC metastasis remain unclear [19].

Our study revealed that TGFβ2 and NDRG1 are upregulated in GC, indicating a poor prognosis. TGFβ2 upregulates NDRG1 transcriptionally, promoting GC metastasis but suppressing the proliferation of GC cells in vitro. TGFβ2 overexpression activates the TGFβR/Smad2/3 pathway, and phosphorylated Smad2/3 promotes NDRG1 expression by binding to its promoter region. Therefore, inhibiting the TGFβ2/Smad2/3‐NDRG1 axis opposes GC progression.

## 2 Results

### 2.1 TGFβ2 is Upregulated in GC Tissue and Shows a Tendency to be Associated With Poor Prognosis

**FIGURE 1:**
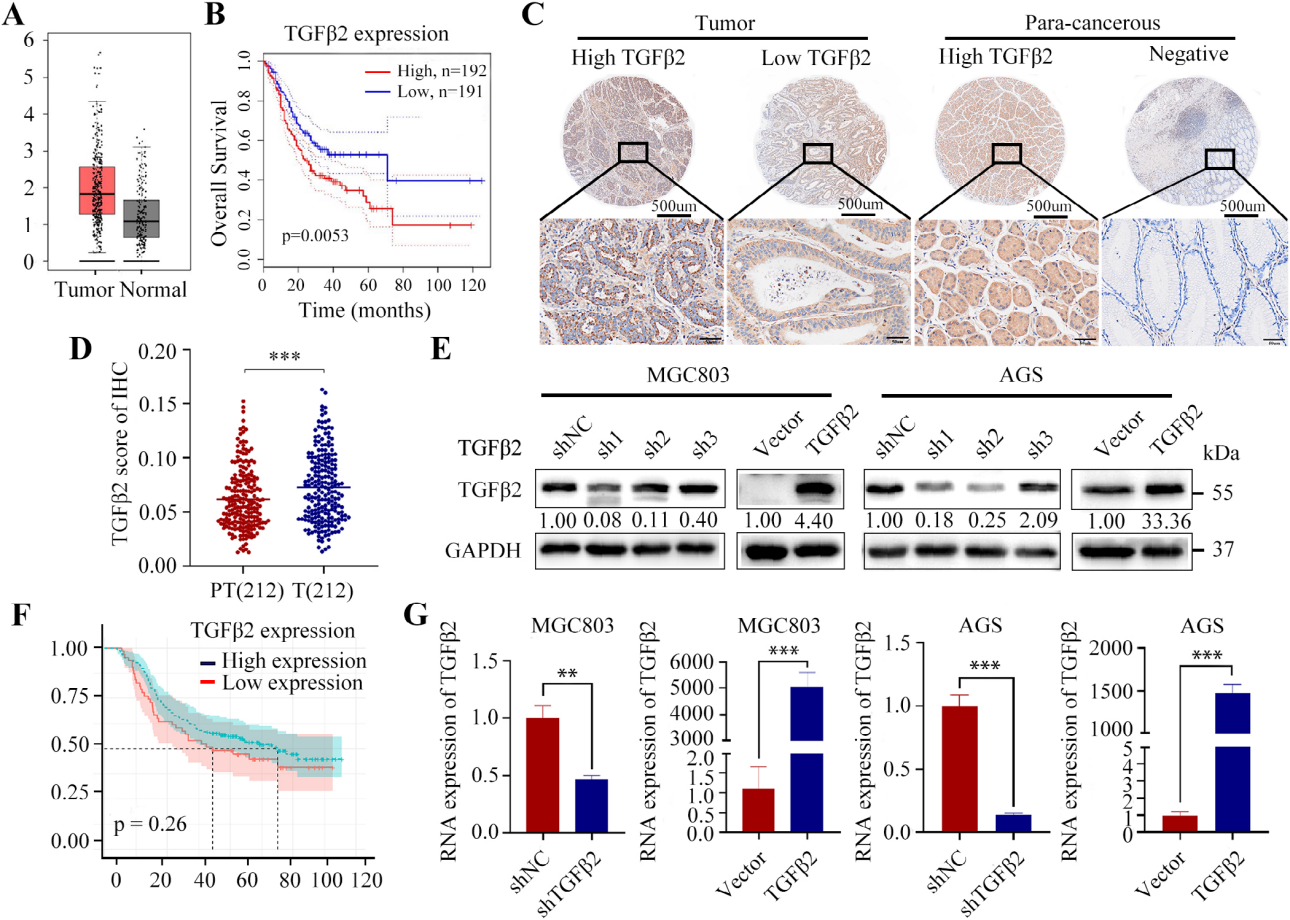
The expression levels of TGFβ2 in gastric cancer tissues and normal tissues were analyzed using data from the TCGA and GTEx databases (A). The relationship between TGFβ2 expression levels and overall survival time was analyzed (B). Representative images of IHC staining in gastric cancer tissues and adjacent noncancerous tissues, using an antibody against TGFβ2, are shown (C). In 212 paired samples, TGFβ2 expression was significantly different between gastric cancer tissues and adjacent noncancerous tissues (D). The effects of stable TGFβ2 interference and overexpression in gastric cancer cell lines were confirmed by Western blot analysis (E). Analysis of gastric cancer samples revealed a significant association between upregulated TGFβ2 expression and 5‐year overall survival in patients with gastric cancer (F). TGFβ2 interference and overexpression were validated at the RNA level (G) (scale bars: 500 and 50 µm). Statistical significance is indicated as follows: **p < 0.01, ***p < 0.001.

To investigate the relevance of TGFβ2 in GC, we analyzed TGFβ2 expression in the GC context via mining the data obtained from The Cancer Genome Atlas (TCGA, https://tcgadata.nci.nih.gov/tcga/) and the Genotype‐Tissue Expression (GTEx, https://gtexportal.org/)., and found that TGFβ2 mRNA levels were significantly upregulated in GC tissues than in normal tissue samples (Figure 1A), and this upregulation is significantly associated with poor prognosis (Figure 1B).

We analyzed TGFβ2 protein expression in GC and adjacent tissues from 212 gastrectomized patients using IHC (Figure 1C). The results showed significant upregulation of TGFβ2 in GC tissues (Figure 1D).

The high‐expression TGFβ2 group had smaller tumors and better differentiation than the low‐expression group. TGFβ2 expression did not correlate significantly with clinical characteristics (Table S1). Although not statistically significant, the high‐expression group tended to have a lower 5‐year survival rate (Figure 1F).

### 2.2 TGFβ2 Promotes GC Cell Metastasis Both In Vitro and In Vivo

**FIGURE 2:**
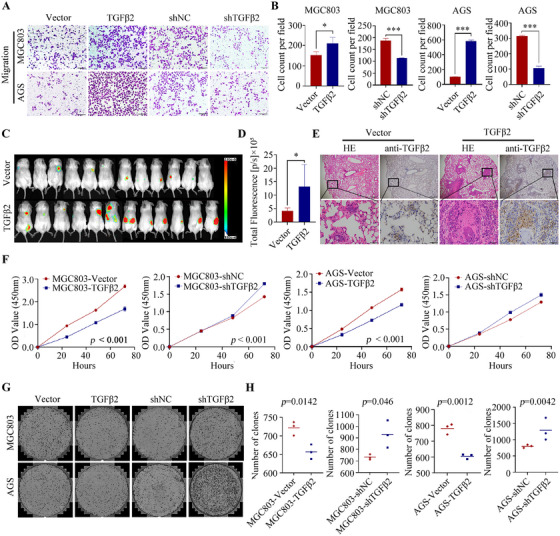
Migration assays of AGS and MGC803 cells with TGFβ2 interference or overexpression were performed (A), and corresponding statistical analyses are shown on the right side (B). The metastatic ability of TGFβ2‐overexpressing MGC803 cells was assessed by in vivo imaging using D‐Luciferin (C), and the corresponding quantitative analysis of fluorescence intensity is shown on the right side (D). TGFβ2 expression was immunohistochemically detected in lung metastatic tumors from mice (E) (scale bars: 500 and 50 µm). The proliferation rates of AGS and MGC803 cells in different treatment groups were measured using the CCK‐8 assay (F). The colony‐formation ability of AGS and MGC803 cells was compared using a plate colony assay in different treatment groups (G), and the corresponding statistical analyses are shown on the right side (H). Statistical significance is indicated as follows: *p < 0.05, ***p < 0.001.

We further investigated the functions of TGFβ2, and TGFβ2 knockdown and overexpression were performed in MGC803 and AGS cell lines using lentiviral vectors, respectively. Transfection efficiency was confirmed by quantitative real‐time polymerase chain reaction (RT‐qPCR) (Figure 1G) and Western blot (Figure 1E). TGFβ2 knockdown significantly reduced the migration ability of MGC803 and AGS cells in vitro (Figure 2A,B), and overexpression of TGFβ2 had the opposite effect (Figure 2A,B). Colony formation assay showed TGFβ2 knockdown promoted MGC803 and AGS cells proliferation, and vice versa (Figure 2F–H). In vivo mouse model showed that overexpression of TGFβ2 promoted the lung metastatic ability of MGC803 cells (Figure 2C–E).

### 2.3 NDRG1 Promotes GC Cell Metastasis Both In Vitro and In Vivo

**FIGURE 3:**
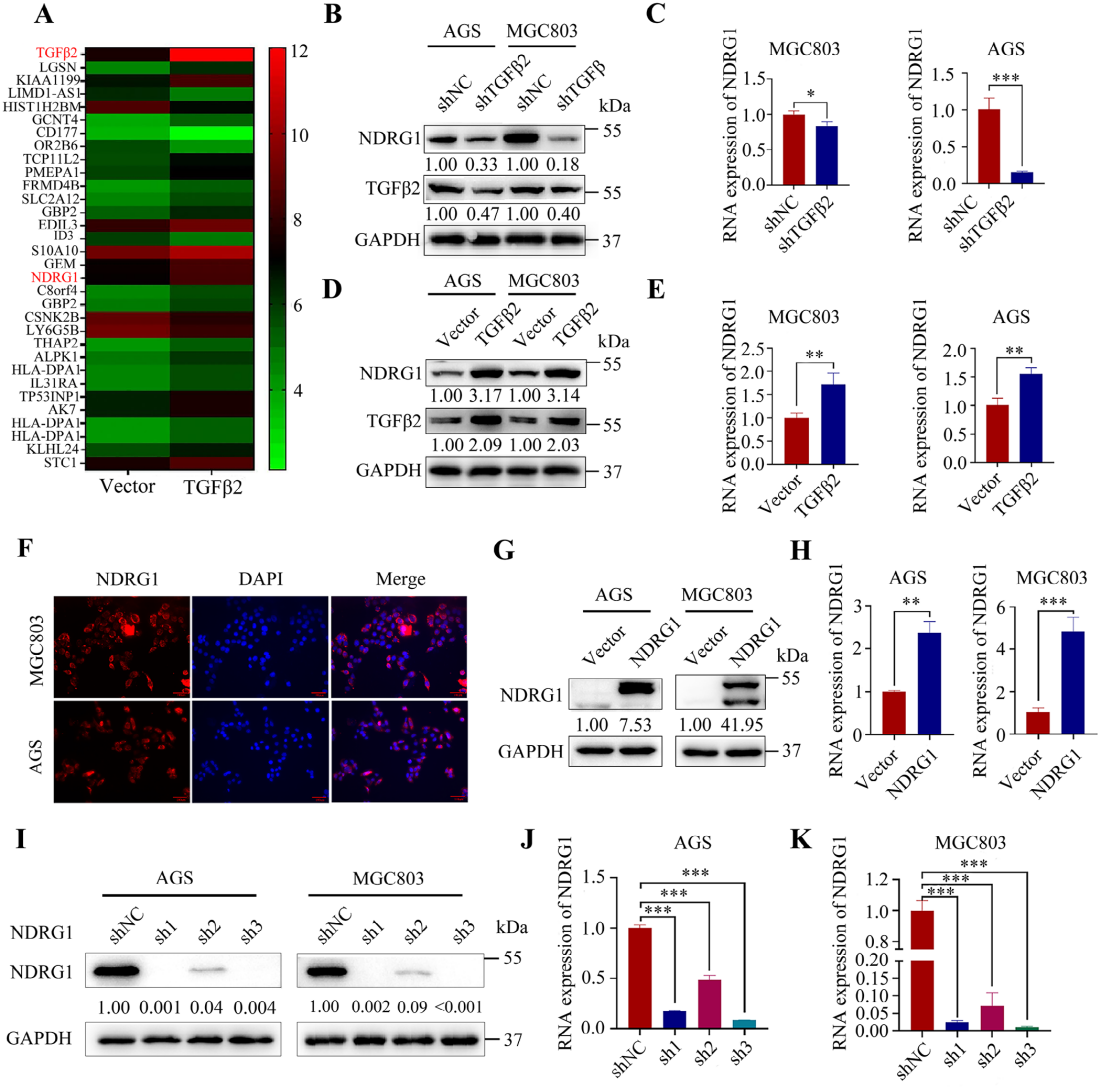
The heatmap shows the top 30 genes with significant expression changes identified by RNA sequencing following TGFβ2 overexpression (A). Western blotting was performed to analyze TGFβ2 and NDRG1 expression in AGS and MGC803 cells, with GAPDH as the loading control (B, D). The mRNA levels of NDRG1 in AGS and MGC803 cells were measured by real‐time qPCR, with GAPDH as the loading control (C, E). NDRG1 (orange) was detected in AGS and MGC803 cells by immunofluorescence staining (F). NDRG1 expression in AGS and MGC803 cells was analyzed by Western blotting and real‐time qPCR, with GAPDH as the loading control (G, H). NDRG1 expression in AGS and MGC803 cells was further confirmed by Western blotting and real‐time qPCR, with GAPDH as the loading control (I–K). Statistical significance is indicated as follows: *p < 0.05, **p < 0.01, ***p < 0.001.

**FIGURE 4:**
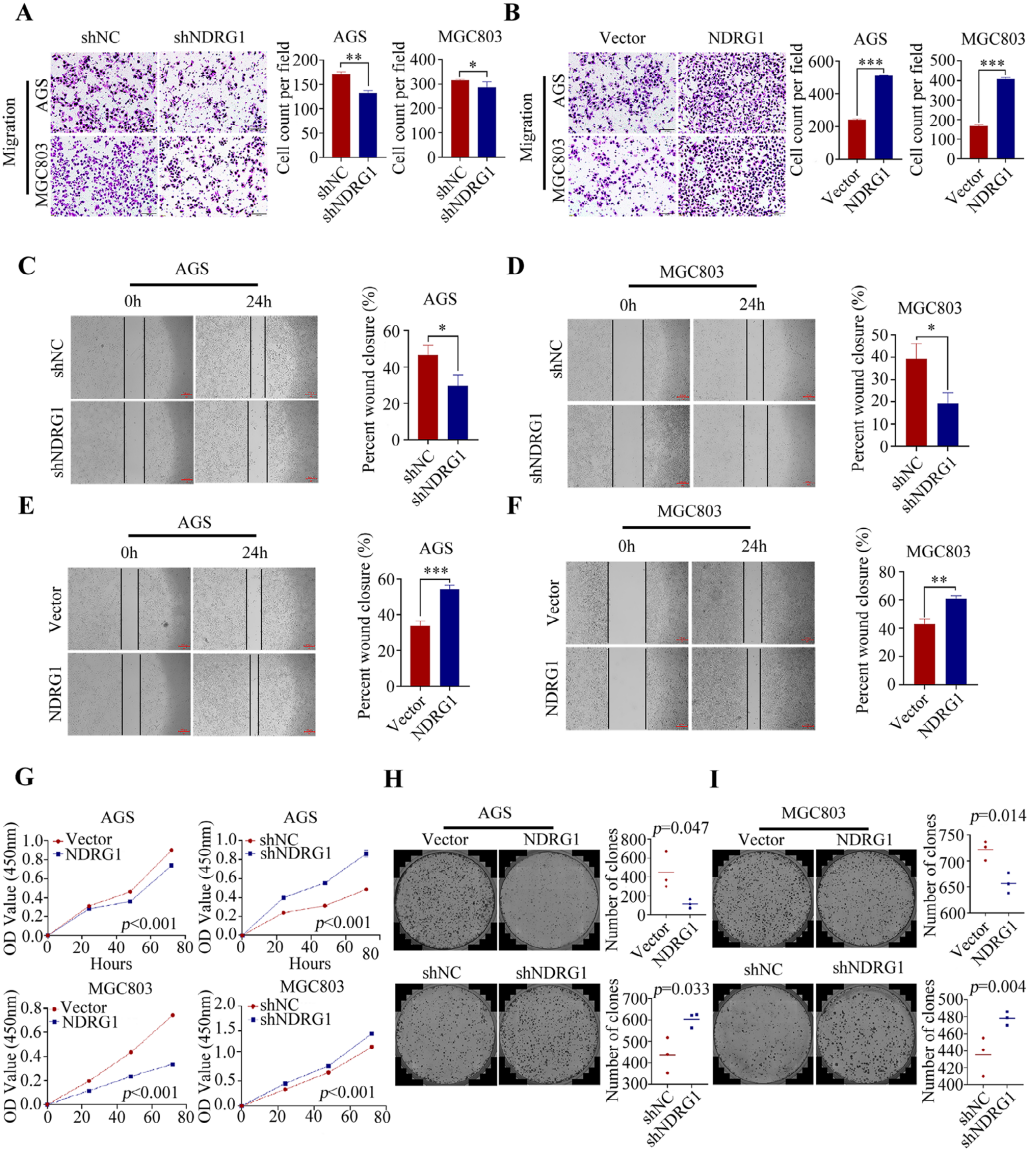
Migration assays were conducted in AGS and MGC803 cells with NDRG1 interference or overexpression, with statistical analyses shown on the right (A, B). Wound healing assays further measured the migratory capacity of these cells under the same conditions (C–F; statistics shown right). Proliferation rates of cells in different treatment groups (control, NDRG1‐interfered, and NDRG1‐overexpressed) were assessed using the CCK‐8 assay (G). Colony formation ability was compared across groups via colony formation assay (H, I; statistics shown right). *p < 0.05, **p < 0.01, ***p < 0.001.

**FIGURE 5:**
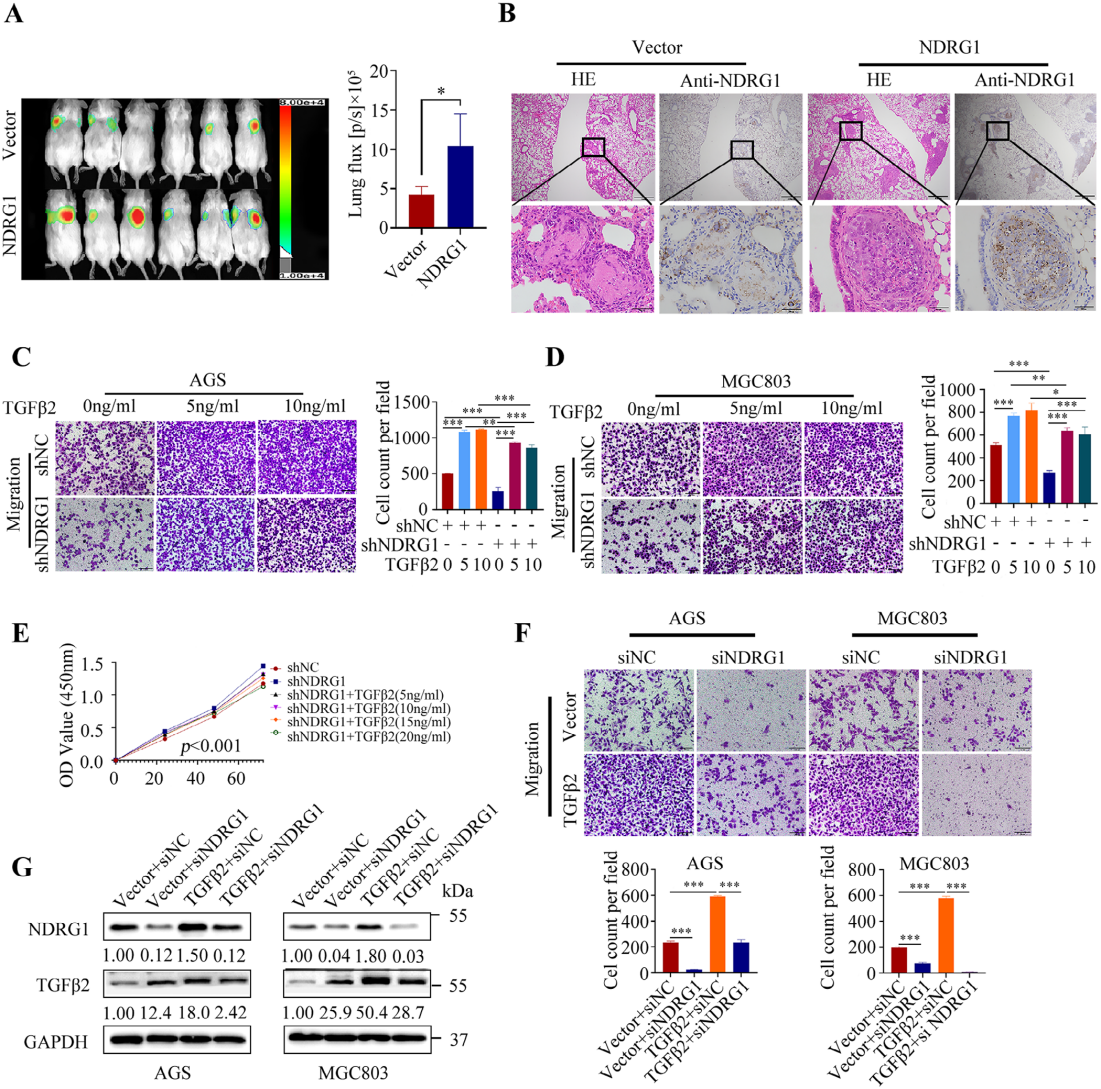
The lung metastatic ability of NDRG1‐overexpressing MGC803 cells was measured by in vivo imaging using D‐Luciferin, and the corresponding quantitative analysis of fluorescence intensity is shown on the right side (A). NDRG1 expression was immunohistochemically detected in lung metastatic tumors from mice (B). The migration assay of AGS and MGC803 cells with NDRG1 expression interference, cocultured with exogenous recombinant TGFβ2 protein, is displayed on the right side (C, D), along with corresponding statistical analyses. As the concentration of exogenous recombinant human TGFβ2 increased, the inhibition of GC cell proliferation became more significant (E). Overexpression of TGFβ2 promoted cell migration and upregulated NDRG1 expression, which was reversed by NDRG1 knockdown (F, G) (scale bars: 500 and 50 µm). Statistical significance is indicated as follows: *p < 0.05, **p < 0.01, ***p < 0.001.

To gain insight into the mechanism of action of TGFβ2 in GC progression, we performed RNA‐seq analysis of the differentially expressed genes in AGS cells with or without TGFβ2 knockdown (Figure 3A). TGFβ2 knockdown downregulated the expression of NDRG1 at both the mRNA and protein levels (Figure 3B–E). Immunofluorescence assay showed that NDRG1 was expressed in cytoplasm, membrane, and nucleus, but mainly in cytoplasm (Figure 3F). However, subcellular localization may be associated with the cellular microenvironment, stress status, and cell type. Study established stable NDRG1‐knockdown and ‐overexpression GC cell lines (Figure 3G–K). NDRG1 knockdown significantly reduced the migration ability of GC cells (Figure 4A,C,D) while enhancing proliferation and colony formation ability (Figure 4G–I), and vice versa (Figure 4B,E–I). Meanwhile, NDRG1 overexpression promoted lung metastasis of GC in mouse models (Figure 5A,B).

### 2.4 GC Cells Migration Promoted by TGFβ2 Overexpression Was Rescued by NDRG1 Interfered

To validate the regulatory effect of TGFβ2 on NDRG1 expression, the study employed different concentrations of exogenous TGFβ2 on GC cells with NDRG1 knockdown. The results demonstrated varying degrees of reversal in the inhibition of migration (Figure 5C,D) and enhancement of proliferation (Figure 5E) caused by NDRG1 knockdown in GC cells. As the concentration of TGFβ2 increased, the inhibition of proliferation became more pronounced, suggesting that the biological effects of TGFβ2 on GC cells may vary depending on its expression levels. Furthermore, the migratory promotion and upregulation of NDRG1 induced by TGFβ2 overexpression were reversed by NDRG1 knockdown (Figure 5F,G).

### 2.5 TGFβ2 Transcriptionally Regulates NDRG1 Expression by Activating the Smad2/3 Signaling Pathway in GC Cells

**FIGURE 6:**
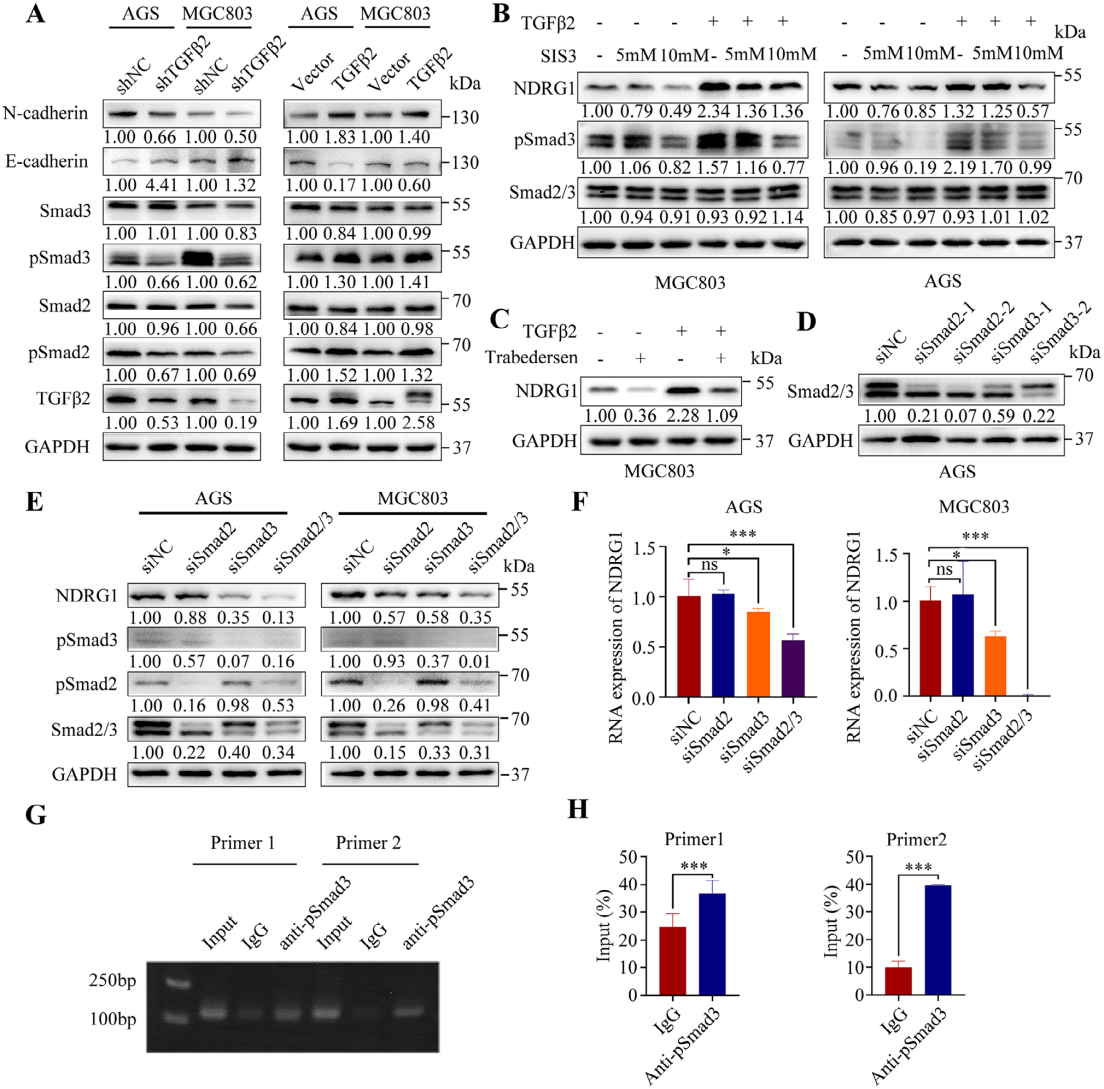
Western blotting of TGFβ2, Smad2/3, pSmad2/3, E‐cadherin, and N‐cadherin expression in AGS and MGC803 cells with TGFβ2 interference or overexpression. GAPDH served as the internal control (A). Effects of SIS3 concentration on NDRG1 expression in AGS and MGC803 cells (B). Effect of TGFβ /Smad inhibitor Trabedersen on NDRG1 expression in MGC803 cells (C). Effect of siRNA interference on Smad2/3 expression (D). Interference of Smad2, Smad3, and SMAD2/3 expression on NDRG1 protein and RNA level in AGS and MGC803 cells (E, F). PCR‐amplified products from ChIP assays of MGC803 cells were detected by Southern blotting. Input acted as a positive control, while IgG served as a negative control (G, H). Statistical significance is indicated as follows: *p < 0.05, ***p < 0.001, ns: not significant.

**FIGURE 7:**
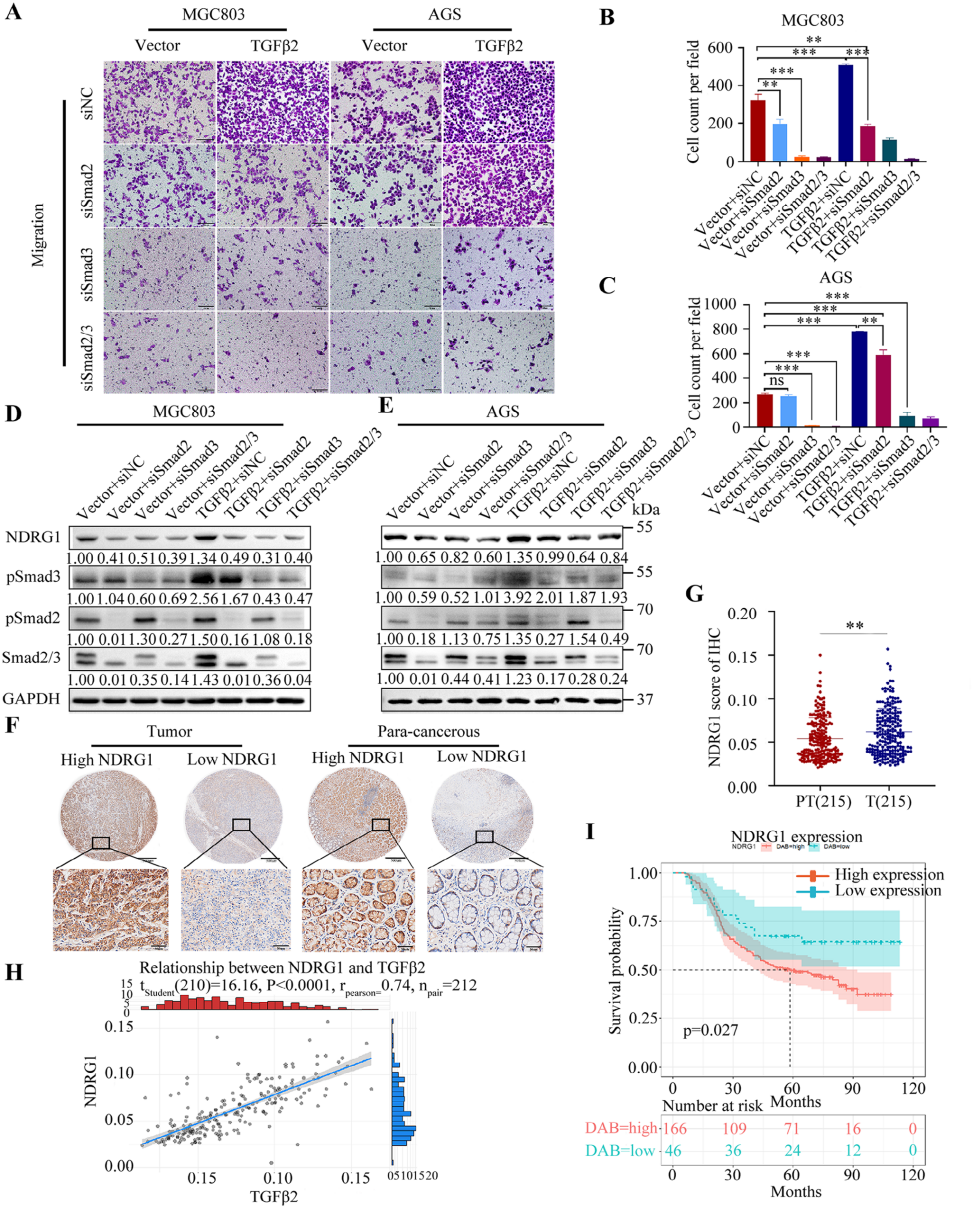
Migration assays of AGS and MGC803 cells following the interference of Smad2, Smad3, or SMAD2/3 expression, respectively, with corresponding statistical analyses shown on the right side (A−C) (scale bars: 100 µm). Western blotting of Smad2/3, pSmad2/3, and NDRG1 expression in AGS and MGC803 cells (D, E). Representative IHC staining for NDRG1 in gastric cancer samples (F) with corresponding statistical analyses (G) shown on the right side (scale bars: 100 and 500 µm). Spearman correlation analysis of IHC staining scores of TGFβ2 and NDRG1: *r* = 0.74, *p* < 0.001 (H). Relationship between NDRG1 expression levels in gastric cancer tissues and patient OS (I): *p* = 0.0027. Statistical significance is indicated as follows: **p < 0.01, ***p < 0.001, ns: not significant.

TGFβ2 knockdown downregulated the phosphorylation levels of Smad2 and Smad3, inhibited the expression of N‐cadherin, but promoted the expression of E‐cadherin (Figure 6A). Similarly, TGFβ2 overexpression yielded opposite results (Figure 6A). Smad3 inhibitor (SIS3) and TGFβ/Smad pathway–specific inhibitor (Trabedersen) consistently inhibited NDRG1 protein level in GC cells (Figure 6B,C). Our study further verified the regulatory relationship between Smad2, Smad3, and NDRG1 by constructing siRNA to interfere with the expression of Smad2, Smad3, and Smad2/3, respectively (Figure 6D). Smad3 knockdown downregulated NDRG1 at both mRNA and protein levels in GC cells (Figure 6E,F), whereas Smad2 knockdown alone did not show significant effects on NDRG1 expression. However, both Smad2 and Smad3 knockdown simultaneously, and the suppression of NDRG1 expression was most pronounced (Figure 6E,F). pSmad2 seemingly enhanced the regulation of NDRG1 by pSmad3. We designed two primers, Primer1 and Primer2, specifically targeting a 2000‐bp nucleotide sequence region located upstream of the transcription start site of NDRG1 (Table S2). The nucleotide fragments enriched by the pSmad3 primary antibody in the chromatin immunoprecipitation (ChIP) assay were subjected to agarose gel electrophoresis, and the result showed that pSmad3 bound to the NDRG1 promoter (Figure 6G). The ChIP‐qPCR assay confirmed that pSmad3 directly bound to the promoter of NDRG1, which transcriptionally regulated NDRG1 expression in GC cells (Figure 6H). Further research found that Smad2, Smad3, or Smad2/3 knockdown reversed TGFβ2 overexpression–induced migration facilitation in vitro (Figure 7A–C) and also reversed TGFβ2 overexpression–induced NDRG1 expression in GC cell lines (Figure 7D,E).

### 2.6 NDRG1 Is Upregulated in GC and Is an Independent Risk Factor for Poor Prognosis

Finally, IHC was performed to detect the expression levels of NDRG1 in 212 pairs of tumors and adjacent noncancerous tissues (Figure 7F). Tissue microarrays (TMAs) stained with NDRG1‐specific antibodies revealed significant upregulation of NDRG1 in GC (Figure 7G). This upregulation was positively correlated with TGFβ2 expression (Figure 7H) and associated with poor prognosis (Figure 7I). Univariate and multivariate analyses further explored the correlation between NDRG1 expression and clinicopathological features (Table S3). Cox multivariate analysis indicated that upregulation of NDRG1 and higher N stage were independent risk factors associated with poor prognosis of GC patients (Tables S4 and S5).

## 3 Discussion

GC metastasis frequently leads to patient mortality, but its mechanisms remain incompletely understood [1, 7]. This study reveals that TGFβ2 promotes GC metastasis, contributing to poor prognosis. Overexpression of TGFβ2 upregulates NDRG1, and their expressions are positively correlated, jointly enhancing GC cell migration in vitro and lung metastasis in mouse models. Additionally, TGFβ2 activates the TGFβR/Smad signaling pathway, promoting Smad2/3 phosphorylation. Inhibition of Smad2/3 expression, its phosphorylation, or TGFβR can abolish the effect of TGFβ2 in promoting NDRG1 expression. Further research shows pSmad3 binds to the NDRG1 promoter, enhancing its expression. Both TGFβ2 and NDRG1 are upregulated in GC tissues and associated with poor prognosis. These findings suggest that TGFβ2 regulates GC metastasis through the TGFβR/Smad‐NDRG1 pathway, potentially serving as a therapeutic target for GC.

TGFβ2 is upregulated in various advanced tumors [20]. Depending on different cell types, cell states, or microenvironments, it can either promote or inhibit programmed cell death [21, 22]. Previous studies have found that TGFβ2 is closely related to proliferation [23], invasion, and migration of GC cells [24], but the molecular mechanisms remain unclear. TCGA‐STAD and GTEx data analysis revealed the significant upregulation of TGFβ2 in GC tissues, which was significantly associated with a poor prognosis. Our study showed the upregulation of TGFβ2 in 212 pairs of GC tissues compared to adjacent noncancerous tissues, with high expression levels observed in the GC tissues. However, high expression of TGFβ2 was not significantly correlated with prognosis. Transcriptional data in TCGA and GTEx databases mainly originate from the North American Caucasian population, and there may be differences in gene expression patterns between different ethnic groups. Additionally, the limited sample size of clinical specimens and the imbalance in sample sizes between the low‐expression and high‐expression groups could potentially impact the statistical outcomes. Lastly, TGFβ2 exhibits dual functions: it promotes GC cell migration while inhibiting proliferation. The study revealed that TGFβ2 can inhibit the proliferation of GC cell lines, which is a crucial biological function of the TGFβ family members. TGFβ inhibits cell proliferation by suppressing cyclin‐dependent kinases (cdks) or downregulating the c‐Myc gene. Many tumor cells evade this inhibitory mechanism [25]. Furthermore, c‐Myc, similar to N‐Myc, has been demonstrated to function as a transcriptional repressor by binding to the NDRG1 promoter region, thereby inhibiting NDRG1 expression [26]. Our study also found that TGFβ2 can promote the migration of GC cells in vivo and in vitro. Specifically, in the early stages of tumorigenesis, TGFβ2 may inhibit tumor growth by suppressing cell proliferation, inducing apoptosis, and inhibiting angiogenesis. However, in the advanced stage, tumor cells may gradually lose their response to tumor‐inhibitory signals of TGFβ2 and instead respond to signals that promote tumor progression, such as those that enhance tumor invasion and metastasis [27, 28, 29, 30].

In our study, Smad2 knockdown alone did not have a significant effect on NDRG1 expression. However, simultaneous knockdown of both Smad2 and Smad3 resulted in the strongest inhibition of NDRG1 expression and migration of GC cells, indicating that Smad2 may cooperate with Smad3 in transcriptional or posttranscriptional regulation. It can also be observed that Smad2 knockdown significantly abolished the enhancement of GC cell migration caused by TGFβ2 overexpression. This may be due to the significantly amplified synergistic regulatory effect of Smad2 at the transcriptional or translational level. Given the low affinity between Smads and their homologous sequences, the binding of DNA‐binding auxiliary factors is often necessary for cooperative action, and these factors can have either promoting or inhibitory effects on TGFβ signal transduction [31].

TGFβ2 is also involved in the EMT process of various malignant tumors, conferring highly invasive migratory abilities to tumor cells [32, 33]. Our study found that upregulation of TGFβ2 expression in GC cells inhibits the epithelial phenotype marker E‐cadherin in GC cells while promoting the mesenchymal phenotype marker N‐cadherin. EMT is a crucial biological process through which tumor cells acquire invasive and migratory abilities. The loss of epithelial phenotype and the acquisition of mesenchymal phenotype being two key steps in this process [34]. However, the specific mechanism by which TGFβ2 promotes EMT in GC cells is still unclear. NDRG1 is often regarded as a potential metastasis suppressor gene, with studies indicating that NDRG1 can inhibit EMT in the initial stages of metastasis, thereby exerting an inhibitory effect on tumor metastasis [35]. However, interactions between NDRG1 and EMT in GC cells still need further study.

The expression of NDRG1 in GC tissue and its relationship with prognosis remains controversial [36]. Our study showed a significant upregulation of NDRG1 in GC tissues, and its expression positively correlated with TGFβ2. Upregulation of NDRG1 expression was associated with better differentiation but also indicated advanced tumor stage and poor prognosis. As a differentiation‐related gene [37, 38], previous studies have found NDRG1 expression in colonic epithelium and well‐differentiated colon cancer cells but not in metastatic colon cancer tissues and cell lines. Furthermore, NDRG1 can induce differentiation and upregulate the expression of E‐cadherin and alkaline phosphatase in metastatic colon cancer cell line SW620. However, NDRG1 is significantly upregulated in low‐to‐moderate differentiated liver cancer cells compared to well‐differentiated cancer cells [39]. NDRG1 expression has been shown to inhibit the invasion and metastasis of prostate cancer, pancreatic cancer, and colon cancer cells [35, 40, 41]. Conversely, upregulation of NDRG1 expression in thyroid cancer and cervical cancer is significantly associated with tumor invasion, metastasis, and poor prognosis [42, 43].

The TGFβ family has been thoroughly studied in solid tumors and immune fibrotic diseases, yielding remarkable outcomes [44]. Currently, TGFβ inhibitors are approved for the treatment of β‐thalassemia and myelodysplastic syndromes, while TGFβR1 inhibitors for advanced solid tumors are in global trials. SHR‐1701, a bifunctional fusion protein targeting PD‐L1 and TGFβRII, enhances T‐cell activation and immune regulation in the tumor microenvironment and is currently in Phase III trials [45]. As a key immunosuppressant, TGFβ holds potential in cancer therapy, but pan‐TGFβ/TGFβR inhibitors are associated with multisystem side effects that challenge clinical safety. Despite amino acid homology [46], TGFβ subtypes differ in their activation mechanisms; TGFβ2 and TGFβ3 have lower thresholds than TGFβ1, and inhibiting them can treat fibrosis while preserving TGFβ1's immunomodulatory function [13]. Exploring these subtypes aids precise drug development, avoids side effects, and benefits clinical therapy.

Our research has revealed that NDRG1 is a downstream effector of TGFβ2 and is significantly associated with the prognosis of GC. This complements TGFβ signaling's role in GC and provides a precise target for antitumor drug development.

Our study has the following limitations: the entire study process was based on GC cell models and mouse models, which may not fully replicate the complex pathological characteristics of human tumor and the multiple antitumor immune mechanisms in the body. To achieve clinical translation, further in‐depth research is still needed. Secondly, there are often cross‐biological interactions, compensation, and regulation among TGFβ subtypes [13]. This study focused on exploring the biological role and mechanism of TGFβ2 in GC, while the interaction between the three subtypes and their biological functions and signaling pathways in the occurrence and development of GC still needs further exploration. Furthermore, our study's clinical aspects need to be broadened to enhance its relevance in translational research. This expansion should encompass an analysis of a larger patient cohort, more exhaustive survival data, extra clinical parameters, and an in‐depth exploration of how these discoveries could potentially shape therapeutic approaches in treating GC.

Additionally, it was found in this study that Smad2 knockdown alone had no significant effect on NDRG1, but both knockdowns of Smad2 and Smad3 could enhance the biological function of Smad3. Our study confirmed that pSmad3 regulates NDRG1 expression by binding to the NDRG1 promoter region, but the mechanism of Smad2 in this process is still unclear. Finally, the study found that TGFβ2 promotes EMT in GC cells, and further in‐depth research is needed to determine the specific mechanism and whether there is an interaction between the pSmad2/3‐NDRG1 signaling pathway and EMT.

In conclusion, these discoveries indicate that TGFβ2 controls the metastatic process in GC via the TGFβR/Smad‐NDRG1 signaling pathway, hinting at its potential use as a target for GC therapy.

## 4 Materials and Methods

### 4.1 Patients

The fresh frozen samples of tumor and tissues adjacent to tumor used in our study were obtained from the Biobank of West China Hospital, which included GC tissues and adjacent noncancerous tissues from GC patients who underwent radical gastrectomy at Gastric Cancer Center of West China Hospital, Sichuan University, between 2009 and 2014. Complete follow‐up information within 5 years was obtained. Patients with gastric stump cancer, stromal tumors, concomitant other malignancies, or those who had received preoperative treatment were excluded. Our study was approved by the Clinical Experimental and Biomedical Ethics Committee, West China Hospital, Sichuan University [2014 Review (82)].

### 4.2 Lentivirus, siRNA, Antibodies, and Reagents

Lentivirus used in this study is listed in Table S6. TGFβ/Smad inhibitor Trabedersen and Smad3 phosphorylation inhibitor SIS3 were purchased from MedChemExpress (Monmouth Junction, NJ, USA). Antibodies used in this study are listed in Table S7. siRNA used in this study is listed in Table S8.

### 4.3 Cell Culture

The human MGC803 and AGS cells used in the study were purchased from the National Collection of Authenticated Cell Cultures (Shanghai, China). The cell lines were authenticated every 6 months by short tandem repeat (STR) analysis. Cells were cultured in RPMI 1640, 1 × (10‐040‐CVRC, Corning) containing 1% penicillin and streptomycin (PYG0016, BOSTER) and 10% fetal bovine serum (FBS, FSD500, ExCell Bio), and maintained in a 5% carbon dioxide cell incubator.

### 4.4 Lentiviral Transfection Experiment

After culturing the cells to logarithmic growth phase, they were digested with trypsin (PYG0015, BOSTER) and diluted to a concentration of 1 × 10^5^ cells/mL, then seeded into a six‐well plate. The volume of virus needed for transfection per well was calculated based on a multiplicity of infection (MOI) of 20. Each well was supplemented with an appropriate volume of lentiviral infection enhancement solution and cultured in a 37°C cell incubator. After 24 h of transfection, the culture medium was replaced. The cells were further cultured for 48 h, and the expression of green fluorescent protein (GFP) was observed under a fluorescence microscope. When the cells reached 70%–80% confluency, they were selected using complete medium containing 4 µg/mL puromycin.

### 4.5 siRNAs Transfection, Recombinant TGFβ2, and Inhibitors Experiments

The siRNA (Beijing Tsingke Biotech Co. Ltd., China) was diluted to 50 µM. The cells were digested, diluted to a concentration of 1 × 10^5^ cells/mL, and seeded into a six‐well cell culture plate for 24 h until the cells were adhered to the wall. The final concentration of siRNA for transfection was 50 nM. The siRNA was diluted in 250 µL Opti‐MEM (31985‐062, Gibco, Thermo Fisher Scientific, Waltham, MA, USA). Lipofectamine 3000 (L3000‐015, Invitrogen, Thermo Fisher Scientific, Waltham, MA, USA) was diluted at a ratio of 5 µL per 250 µL Opti‐MEM and was stored at room temperature for 5 min. After mixing the diluted siRNA with diluted Lipofectamine 3000, stored at room temperature for 20 min, the mixture was added to the six‐well cell culture plate. Smad3 inhibitor SIS3 (HY‐13013, MedChemExpress) was dissolved in DMSO (PYG0040, BOSTER) and the mixture was added to the culture dishes to achieve final concentrations of 0, 5, and 10 µM. The cells were incubated in the cell culture incubator for 24 h. Tumor cell treatment was performed in a full culture medium with10 µM Trabedersen (HY‐142118, MedChemExpress) for 7 days. Recombinant human TGFβ2 protein (HZ‐1092, Proteintech) was dissolved in sterile water and the mixture was added to the cell culture dishes in gradient concentrations to achieve final concentrations of 0, 5, 10, 15, and 20 ng/mL. The cell culture was incubated in an incubator at 37°C for 24–48 h.

### 4.6 RNA Isolation and RT‐qPCR Analysis

This study used the FastPure Cell/Tissue Total RNA Isolation kit (RC112‐01, Vazyme) to extract cellular RNA according to the manufacturer's instructions. Reverse transcription polymerase chain reaction (RT‐PCR) and RT‐qPCR were performed using the ExonScript RT SuperMix with dsNDase kit (A502‐02, EXONGEN) following the manufacturer's protocol. Each sample was repeated at least three times, and the results were analyzed by calculating ΔΔ*ct* values. The primers used in the study are listed in Table S2.

### 4.7 Western Blotting

Samples were lysed using RIPA lysis buffer (P0013B, Beyotime) containing a complete protease and phosphatase inhibitor (A32961, Thermo Fisher Scientific, Waltham, MA, USA). The protein concentration was determined using the BCA protein assay kit (CW0014S, CWBIO). Equal amounts of samples were loaded onto SDS‐polyacrylamide gels (PG112, Epizyme) for electrophoresis and transferred to a polyvinylidene difluoride (PVDF) membrane (ISEQ00010, Merck KGaA, Darmstadt, Germany). The membrane was blocked with 5% milk diluted in Phosphate Buffered Saline/Tween (PBST) or Tris‐Buffered Saline/Tween (TBST), then incubated with primary antibody overnight at 4°C followed by secondary antibody incubation for 1 h. Finally, the membrane was detected using Enhanced Chemiluminescent kit (P10200, NCM Biotech).

### 4.8 Transwell Experiment and Wound Healing Assays

The study utilized Transwell Permeable Supports (3422, Corning) for migration assays, with each group repeated at least three times. Cells were suspended in 1640 medium without FBS and adjusted to a density of 3 × 10^4^ cells per 100 µL in each insert. The lower chamber was filled with 600 mL complete medium containing 10% FBS. After incubation for 24 h at 37°C in a cell incubator, the migrated cells were fixed and stained with the Wright‐Giemsa Stain kit (D010, Nanjing Jiancheng Bioengineering Institute, China), and then were observed under a microscope and randomly photographed. For wound healing assays, tumor cells of the same density were seeded into six‐well plates, with each sample repeated three times. Samples were cultivated until the cells reached about 80% confluence, then deprived of serum for 24 h. A 100 µL pipette tip was used to scratch the plates, and then these plates were photographed at 0 and 24 h.

### 4.9 Cell Counting Kit‐8 Assay

The concentration was adjusted to 3 × 10^5^ cells/mL, and 100 µL per well of 96‐well plate was added, each sample was repeated six times. After cell adhesion, at 0, 24, 48, and 72 h time points, under light‐protected conditions, the CCK‐8 reagent (C6005M, UElandy) was mixed with serum‐free culture medium at a volume ratio of 1:9, and then added at a rate of 100 µL per well into the 96‐well plate. It was incubated at 37°C under light‐protected conditions for 2 h, followed by measuring the absorbance at 450 nm.

### 4.10 Immunofluorescence

Slides were placed at the bottom of a 24‐well plate, the cell density was adjusted to 1 × 10^4^ cells per mL (1 mL per well), and slides were then cultured at 37°C in cell incubator for 24 h. These slides were fixed with 4% paraformaldehyde (BL539A, Biosharp) for 30 min, then blocked with PBST containing 5% bovine serum albumin (BSA, 4240, BioFroxx) and 0.3% Triton‐X‐ 100 (H5141, Promega). These slides were incubated with primary antibodies overnight at 4°C (anti‐TGFβ2, 1:200, 19999‐1‐AP, Proteintech, China; anti‐NDRG1, 1:200, ab124689, Abcam, USA), followed by incubation with fluorescent secondary antibodies (1:800, A21428, Thermo Fisher Scientific, Waltham, MA, USA) for 1 h under light‐protected conditions. Slides were stained with DAPI (C0065, Solarbio Life Science, Beijing, China) for 2 min and mounted with a Fluoromount‐G (0100‐01, SouthernBiotech).

### 4.11 Clone Formation Assay

The cell density in the six‐well plate was adjusted to 1 × 10^3^ cells per well, with each sample repeated three times. The cells were incubated at 37°C in a cell incubator for 7–14 days. The cells were stained with crystal violet (548‐62‐9, Look Chem, Shanghai, China) dye for 15 min, followed by washing with water, and scanned and analyzed using the Celigo full‐field cell scanning analyzer (200‐BFFL‐5C, WeiChilab, Shanghai, China).

### 4.12 ChIP Assay

ChIP assays utilized the Pierce Magnetic ChIP kit (26157, Thermo Fisher Scientific, Waltham, MA, USA). Chromatin–protein complexes were cross‐linked with 1% formaldehyde for 10 min, followed by quenching with glycine. After repeated washing with PBS, cells were collected and resuspended in membrane extraction buffer, then incubated on ice for 10 min before centrifugation. Subsequently, the pellet was resuspended in 200 µL of MNase. A total of 1 µL of MNase (10 U/µL) was diluted tenfold, then 6 µL of MNase was added to the cell lysate, mixed thoroughly, and incubated at 37°C for 15 min. Digestion was terminated with 20 µL of MNase Stop Solution. After centrifugation, the pellet was collected and resuspended in MNase Stop Solution (containing PPI). Cell lysis was achieved by sonicating on ice. Next, samples were mixed with pSmad3 antibody or IgG and incubated overnight at 4°C. The next day, Protein A/G Magnetic Beads were added separately to the anti‐pSmad3 and IgG groups and incubated with gentle agitation at 4°C for 2 h. Subsequently, protein/DNA complexes were washed with elution buffer and shaken at 65°C for 30 min. They were then incubated at 65°C with 5 M NaCl and 20 mg/mL Proteinase K for 1.5 h. Finally, DNA fragments were purified using a DNA extraction kit and amplified by qPCR. Primers and primary antibodies are detailed in Tables S2 and S7.

### 4.13 Agarose Gel Electrophoresis

A total of 1% agarose gels was prepared using 1X TAE buffer and an appropriate amount of 10,000X GelRed was added (GR501‐01, Vazyme). An appropriate ratio of loading buffer was added to samples, mixed well, and loaded onto the gel. Electrophoresis was performed at a constant voltage of 140–160 V. Finally, images of gels were captured.

### 4.14 Preparation of TMA and IHC Staining

The paraffin‐embedded blocks intended for TMA construction were routinely sectioned and stained with H&E. The boundaries of tumor and adjacent noncancerous tissues were marked under an optical microscope. The marked regions were then extracted from the paraffin blocks (diameter 1.0 mm) and embedded into new paraffin blocks to create TMAs. The TMA slices underwent deparaffinization and rehydration in xylene, ethanol, and deionized water. Subsequently, the slices were heated in 0.01 M sodium citrate buffer and naturally cooled for 20 min for antigen retrieval. Endogenous peroxidase was removed by incubating the slices with 3% hydrogen peroxide for 20 min. Next, the TMA slices were incubated with the primary antibody overnight at 4°C, followed by incubation with Donkey anti‐Rabbit IgG (KIT‐9710, MXB Biotechnologies) for 1 h. The slices were then stained with diaminobenzidine (DAB) and counterstained with hematoxylin. After dehydration in ethanol and xylene, the slices were mounted.

### 4.15 Quantitative Analysis of IHC Staining Results

The research utilized QuPath‐0.5.1 software for the quantitative analysis of IHC staining results.

1. The mean staining intensity of 3,3′‐DAB was used as the quantification standard (Table S9). The IHC staining areas were selected using the “TMAdearrayer” option under the TMA menu, with the option to manually add any areas not automatically recognized by the software.
2. DAB mean intensity was calculated using the calculate feature—“Add intensity feature” under the Analyze menu.
3. The stained areas were selected and TMA cores were performed for analysis.
4. Finally, the analysis results were exported for further analysis using “Export measurements” option under the Measure menu.

### 4.16 Animal Experiments

The human GC cells MGC803, stably transfected with TGFβ2 or NDRG1 (2 × 10^6^ cells/injection), were injected into the tail vein of 5‐week‐old male NCG mice, targeting the distal 1/3 to 2/3 of the vein. Fluorescence intensity within the mice was observed weekly, and fluorescence intensity was assessed and photographed in the fourth week post injection. Lung tissues were collected and fixed in formalin for histological analysis. Animal experiments were approved by the Ethics Committee for Laboratory Animals of West China Hospital, Sichuan University (Ethic record No.: 20230426007).

### 4.17 Statistical Analysis

This study used ImageJ 1.53t (Wayne Rasband and contributors National Institutes of Health, USA), GraphPad Prism 8.0 (GraphPad Inc., San Diego, CA, USA), and R 4.2. 1 for data organization, analysis, and graphing. Two independent‐sample *t*‐tests were used for intergroup comparisons. Continuous variables were described using mean ± standard deviation (Mean ± SD). Pearson's chi‐square test was used for comparison of categorical variables. Kaplan–Meier method was used for survival analysis and survival curve plotting. We determined the optimal cutoff value for the mean DAB intensity using the survminer R package. Cox regression model was used for multifactor analysis of prognostic‐related risks. *p* < 0.05 was considered statistically significant (*p < 0.05, **p < 0.01, ***p < 0.001).

## Author Contributions

F.‐J.H., X.‐L.C., and Y.‐F.Z. conceived and designed the experiments, performed the experiments and collected the data, analyzed and interpreted the data, wrote the initial draft of the manuscript, and revised the manuscript critically for important intellectual content. Y.‐F.Z., Z.‐D.L., P.‐P.L., and H.‐Y.P. contributed to the conception and design of the experiments, provided assistance in data collection and analysis, contributed to the writing, and approved the final version of the manuscript to be published. T.J., Z.‐W.C., and Z.‐H.C. contributed to the acquisition of data and the analysis and interpretation of the data. J.‐K.H. and K.Y. oversaw the entire research project and provided guidance and supervision throughout the experimental process, contributed significantly to the interpretation of the results and critically reviewed and edited the manuscript, and ensured that all authors approved the final version of the manuscript and communicated with the journal editors and reviewers during the publication process. All authors have read and approved the final manuscript and agree to be accountable for all aspects of the work in ensuring that questions related to the accuracy or integrity of any part of the work are appropriately investigated and resolved.

## Ethics Statement

This study was approved by the Ethics Committee for Laboratory Animals of West China Hospital, Sichuan University (20230426007) and the Clinical Experimental and Biomedical Ethics Committee, West China Hospital, Sichuan University [2014 Review (82)].

## Conflicts of Interest

The authors declare no conflicts of interest.

## Supporting information



Supporting Information

## Data Availability

Clinical data for the gastric cancer cohort analyzed in this study were obtained from the electronic medical record system of West China Hospital Sichuan University, with longitudinal follow‐up records retrieved from the institution's prospectively maintained gastric cancer registry. The postoperative surveillance protocol mandated clinical evaluations at 3‐ to 6‐month intervals during the initial 24‐month period, transitioning to biannual or annual assessments commencing from the third postoperative year. In compliance with institutional review board regulations and patient confidentiality agreements, the raw datasets generated during this investigation are not publicly available.
