# Supplementary material for: Transforming Growth Factor Beta2 Promotes Migration and Inhibits the Proliferation of Gastric Cancer Cells by Regulating the pSmad2/3‐NDRG1 Signaling Pathway
Source: MedComm (2020). 2025 Mar 27;6(4):e70148. doi: 10.1002/mco2.70148 (PMC11949502; doi:10.1002/mco2.70148)
Supplement: Supplementary file 1 — Supporting Information [file MCO2-6-e70148-s001.docx]

| **Supplementary Table 1. TGFβ2 expression and clinicopathology of GC** | | | | |
| --- | --- | --- | --- | --- |
|  |  | TGFβ2 | |  |
|  |  | Low-expression | High-expression |  |
| Variables | | n=62(%) | n=148(%) | P value |
| Gender | Male | 44 (71.0) | 113 (76.4) | 0.413 |
|  | Female | 18 (29.0) | 35 (23.6) |  |
| Age (years) | ≧65 | 23 (37.1) | 61 (41.2) | 0.578 |
|  | <65 | 39 (62.9) | 87 (58.8) |  |
| Tumor site | Upper | 15 (24.2) | 48 (32.4) | 0.493 |
|  | Middle | 11 (17.7) | 23 (15.5) |  |
|  | Low | 36 (58.1) | 77 (52.0) |  |
| Tumor size | ≧5cm | 45 (72.6) | 118 (79.7) | 0.257 |
|  | <5cm | 17 (27.4) | 30 (20.3 ) |  |
| Bormann classification | Bormann I | 3 (4.8) | 7 (4.7 ) | 0.229 |
|  | Bormann II | 28 (45.2) | 62 (41.9) |  |
|  | Bormann III | 23 (37.1) | 68 (45.9) |  |
|  | Bormann IV | 8 (12.9) | 11 (7.4) |  |
| Differentiation | Differentiated | 18 (29.0) | 50 (33.8) | 0.502 |
|  | Undifferentiated | 44 (71.0) | 98 (66.2) |  |
| T Stage | T1 | 4 (6.5) | 4 (2.7) | 0.612 |
|  | T2 | 7 (11.3) | 20 (13.5) |  |
|  | T3 | 13 (21.0) | 33 (22.3) |  |
|  | T4 | 38 (61.3) | 91 (61.5) |  |
| N Stage | N0 | 13 (21.0) | 39 (26.4) | 0.201 |
|  | N1 | 8 (12.9) | 34 (23.0) |  |
|  | N2 | 13 (21.0) | 23 (15.5) |  |
|  | N3 | 28 (45.2) | 52 (35.1) |  |
| TNM Stage | I | 8 (12.9) | 12 (8.1) | 0.095 |
|  | II | 9 (14.5) | 41 (27.7) |  |
|  | III | 45 (72.6) | 95 (64.2) |  |
| 5-OS | - | 56.4±6.0 | 43.8±4.2 | 0.280 |
| Note：The degree of differentiation of GC is referred to the sixth edition of the Japanese Gastric Cancer Diagnosis and Treatment Guide [46]. GC: Gastric cancer. OS: Overall survival. | | | | |

| **Supplementary Table 2. Primers used in the study** | | | |
| --- | --- | --- | --- |
| Gene | Species | Forward/Reverse primer | Reverse primer (5’－3’) |
| TGFβ2 | Human | F | GCGCTACATCGACAGCAAAG |
|  |  | R | TGCAGCAGGGACAGTGTAAG |
| NDRG1 | Human | F | CATACTGCGCCTAACTCGGT |
|  |  | R | GGGCACCCACGTAATAGACC |
| NDRG1-promoter1 | Human | F | GGTGGGGGAATGTGCTCCTA |
|  |  | R | TCCAGTGTCATGGGTTTTGC |
| NDRG1-promoter2 | Human | F | GGGGGAATGTGCTCCTACAT |
|  |  | R | TCCAGTGTCATGGGTTTTGC |
| GAPDH | Human | F | GGTGGAATCATATTGGAACA |
|  |  | R | CTCTGGTAAAGTGGATATTGT |
| KIAA1199 | Human | F | CTCTGGTAAAGTGGATATTGT |
|  |  | R | GGTGGAATCATATTGGAACA |

| **Supplementary Table 3. NDRG1 expression and clinicopathology of GC** | | | | |
| --- | --- | --- | --- | --- |
|  |  | NDRG1 | |  |
|  |  | Low-expression | High-expression |  |
| Variables | | n=68(%) | n=147(%) | P value |
| Gender | Male | 51 (75.0) | 110 (74.8) | 0.979 |
|  | Female | 17 (25.0) | 37 (25.2) |  |
| Age (years) | ≧65 | 25 (36.8) | 62 (42.3) | 0.452 |
|  | <65 | 43 (63.2) | 85 (57.7) |  |
| Tumor site | Upper | 21 (30.9) | 48 (32.7) | 0.243 |
|  | Middle | 9 (13.2) | 32 (21.8) |  |
|  | Lower | 38 (55.9) | 67 (45.6) |  |
| Tumor size | ≧5cm | 56 (82.4) | 112 (76.2) | 0.309 |
|  | <5cm | 12 (17.6) | 35 (23.8) |  |
| Bormann classification | Bormann I | 2 (2.9) | 5 (3.4) | 0.6 |
|  | Bormann II | 29 (42.6) | 62 (42.2) |  |
|  | Bormann III | 31 (45.6) | 67 (45.6) |  |
|  | Bormann IV | 6 (8.9) | 13 (8.8) |  |
| Differentiation | Differentiated | 15 (22.1) | 55 (37.4) | **0.025** |
|  | Undifferentiated | 53 (77.9) | 92 (62.6) |  |
| T stage | T1 | 2 (2.9) | 6 (4.1) | 0.953 |
|  | T2 | 8 (11.8) | 20 (13.6) |  |
|  | T3 | 15 (22.1) | 31 (21.1) |  |
|  | T4 | 43 (63.2) | 90 (61.2) | 0.168 |
| N stage | N0 | 13 (19.1) | 41 (27.9) |  |
|  | N1 | 11 (16.2) | 32 (21.8) |  |
|  | N2 | 15 (22.1) | 22 (15.0) |  |
|  | N3 | 29 (42.6) | 52 (35.4) |  |
| TNM stage | I | 7 (10.3) | 14 (9.5) | **0.024** |
|  | II | 8 (11.8) | 42 (28.6) |  |
|  | III | 53 (77.9) | 91 (61.9) |  |
| 5-year OS | - | 59.7±6.7 | 36.2±5.4 | **0.026** |
| Note：The degree of differentiation of GC is referred to the sixth edition of the Japanese Gastric Cancer Diagnosis and Treatment Guide [46]. GC: Gastric cancer. OS: Overall survival. | | | | |

| **Supplementary Table 4. Univariate analysis of prognostic correlation in GC** | | |
| --- | --- | --- |
| Variables | 95%CI | P value |
| Gender | 0.363 ─ -1.662 | 0.097 |
| Age | 0.333 ─ 1.659 | 0.097 |
| Tumor size | 0.436 ─ -1.487 | 0.065 |
| Tumor site | 0.178 ─ -2.177 | **0.029** |
| Differentiation | 0.353 ─ 1.536 | 0.124 |
| T stage | 0.273 ─ 1.467 | 0.142 |
| Nstage | 0.207 ─ 2.636 | **0.008** |
| Bormann classification | 0.249 ─ 0.325 | 0.745 |
| NDRG1 expression | 0.346 ─ -2.584 | **0.009** |
| OR: odds ratio. CI: confidence interval. GC: Gastric cancer. | | |

| **Supplementary Table 5. Multivariate analysis of prognostic correlation in GC** | | | |
| --- | --- | --- | --- |
| Variables | OR | 95%CI | P value |
| Gender | 0.556 | 0.272 - 1.118 | 0.102 |
| Age | 1.642 | 0.882 - 3.100 | 0.121 |
| Tumor size | 0.464 | 0.198 - 1.043 | 0.068 |
| T stage | 1.635 | 1.088 - 2.519 | **0.021** |
| N stage | 1.892 | 1.444 - 2.523 | **<0.001** |
| Tumor site | 0.705 | 0.499 - 0.989 | **0.045** |
| NDRG1 expression | 0.440 | 0.223 - 0.846 | **0.015** |
| OR: odds ratio. CI: confidence interval. GC: Gastric cancer. | | | |

| **Supplementary Table 6. Lentivirus information used in the study** | | | | |
| --- | --- | --- | --- | --- |
| Gene | Target Sequence | | CDS | GC% |
| TGFβ2-RNAi | CGGATTGAGCTATATCAGATT | | 1367..2611 | 31.58% |
| NDRG1-RNAi | TGCTCTAAACAACCCTGAGAT | | 138..1322 | 42.11% |
| LV-TGFβ2 | GTTTTTGGCTTTTTTGTTAGACGAAGCTTGGGCTGCAGGTCGACTCTAGAGGATCCCGCCACCATGCACTACTGTGTGCTGAGCGCTTTTCTGATCCTGCATCTGGTCACGGTCGCGCTCAGCCTGTCTACCTGCAGCACACTCGATATGGACCAGTTCATGCGCAAGAGGATCGAGGCGATCCGCGGGCAGATCCTGAGCAAGCTGAAGCTCACCAGTCCCCCAGAAGACTATCCTGAGCCCGAGGAAGTCCCCCCGGAGGTGATTTCCATCTACAACAGCACCAGGGACTTGCTCCAGGAGAAGGCGAGCCGGAGGGCGGCCGCCTGCGAGCGCGAGAGGAGCGACGAAGAGTACTACGCCAAGGAGGTTTACAAAATAGACATGCCGCCCTTCTTCCCCTCCGAAAATGCCATCCCGCCCACTTTCTACAGACCCTACTTCAGAATTGTTCGATTTGACGTCTCAGCAATGGAGAAGAATGCTTCCAATTTGGTGAAAGCAGAGTTCAGAGTCTTTCGTTTGCAGAACCCAAAAGCCAGAGTGCCTGAACAACGGATTGAGCTATATCAGATTCTCAAGTCCAAAGATTTAACATCTCCAACCCAGCGCTACATCGACAGCAAAGTTGTGAAAACAAGAGCAGAAGGCGAATGGCTCTCCTTCGATGTAACTGATGCTGTTCATGAATGGCTTCACCATAAAGACAGGAACCTGGGATTTAAAATAAGCTTACACTGTCCCTGCTGCACTTTTGTACCATCTAATAATTACATCATCCCAAATAAAAGTGAAGAACTAGAAGCAAGATTTGCAGGTATTGATGGCACCTCCACATATACCAGTGGTGATCAGAAAACTATAAAGTCCACTAGGAAAAAAAACAGTGGGAAGACCCCACATCTCCTGCTAATGTTATTGCCCTCCTACAGACTTGAGTCACAACAGACCAACCGGCGGAAGAAGCGTGCTTTGGATGCGGCCTATTGCTTTAGAAATGTGCAGGATAATTGCTGCCTACGTCCACTTTACATTGATTTCAAGAGGGATCTAGGGTGGAAATGGATACACGAACCCAAAGGGTACAATGCCAACTTCTGTGCTGGAGCATGCCCGTATTTATGGAGTTCAGACACTCAGCACAGCAGGGTCCTGAGCTTATATAATACCATAAATCCAGAAGCATCTGCTTCTCCTTGCTGCGTGTCCCAAGATTTAGAACCTCTAACCATTCTCTACTACATTGGCAAAACACCCAAGATTGAACAGCTTTCTAATATGATTGTAAAGTCTTGCAAATGCAGCGGTATGGACTACAAGGATGACGA | |  |  |
| LV-NDRG1 | TTTTTGGCTTTTTTGTTAGACGAAGCTTGGGCTGCAGGTCGACTCTAGAGGATCCCGCCACCATGTCTCGGGAGATGCAGGATGTAGACCTCGCTGAGGTGAAGCCTTTGGTGGAGAAAGGGGAGACCATCACCGGCCTCCTGCAAGAGTTTGATGTCCAGGAGCAGGACATCGAGACTTTACATGGCTCTGTTCACGTCACGCTGTGTGGGACTCCCAAGGGAAACCGGCCTGTCATCCTCACCTACCATGACATCGGCATGAACCACAAAACCTGCTACAACCCCCTCTTCAACTACGAGGACATGCAGGAGATCACCCAGCACTTTGCCGTCTGCCACGTGGACGCCCCTGGCCAGCAGGACGGCGCAGCCTCCTTCCCCGCAGGGTACATGTACCCCTCCATGGATCAGCTGGCTGAAATGCTTCCTGGAGTCCTTCAACAGTTTGGGCTGAAAAGCATTATTGGCATGGGAACAGGAGCAGGCGCCTACATCCTAACTCGATTTGCTCTAAACAACCCTGAGATGGTGGAGGGCCTTGTCCTTATCAACGTGAACCCTTGTGCGGAAGGCTGGATGGACTGGGCCGCCTCCAAGATCTCAGGATGGACCCAAGCTCTGCCGGACATGGTGGTGTCCCACCTTTTTGGGAAGGAAGAAATGCAGAGTAACGTGGAAGTGGTTCACACCTACCGCCAGCACATTGTGAATGACATGAACCCCGGCAACCTGCACCTGTTCATCAATGCCTACAACAGCCGGCGCGACCTGGAGATTGAGCGACCAATGCCGGGAACCCACACAGTCACCCTGCAGTGCCCTGCTCTGTTGGTGGTTGGGGACAGCTCGCCTGCAGTGGATGCCGTGGTGGAGTGCAACTCAAAATTGGACCCAACAAAGACCACTCTCCTCAAGATGGCGGACTGTGGCGGCCTCCCGCAGATCTCCCAGCCGGCCAAGCTCGCTGAGGCCTTCAAGTACTTCGTGCAGGGCATGGGATACATGCCCTCGGCTAGCATGACCCGCCTGATGCGGTCCCGCACAGCCTCTGGTTCCAGCGTCACTTCTCTGGATGGCACCCGCAGCCGCTCCCACACCAGCGAGGGCACCCGAAGCCGCTCCCACACCAGCGAGGGCACCCGCAGCCGCTCGCACACCAGCGAGGGGGCCCACCTGGACATCACCCCCAACTCGGGTGCTGCTGGGAACAGCGCCGGGCCCAAGTCCATGGAGGTCTCCTGCGGTATGGACTACAAGGATGACGATGACAAGGATTACAAAGACGACGATGATAAGGACTATAAGGATGATGACGACAAATGAGCTAGCACATAACTTACGGTAAATGGCCCGCCTGGCTGACCGCCCAACGACCCCCGCCCATTGACGTCAATAGTAACGCCAATAGGGACTTTCCATTGACGTCAATGGGTGGAGTATTTACGGTAAACTGCCCACTTGGCAGTACATCAAGTGTATCATATGCCAAGTACGCCCCCTATTGACGTCAATGACGGTAAATGGCCCGCCTGGC | |  |  |
|  | | Vector | | |
| GV542 Luciferase Neomycin | | pGC-FU-firefly_Luciferase-SV40-neomycin | | |
| TGFβ2-RNAi | | pGC-FU-3FLAG-CBh-gcGFP-IRES-puromycin | | |
| LV-TGFβ2 | | pGC-FU-3FLAG-CBh-gcGFP-IRES-puromycin | | |
| NDRG1-RNAi | | pFU-GW-016 | | |
| LV-NDRG1 | | pGC-FU-3FLAG-CBh-gcGFP-IRES-puromycin | | |

| **Supplementary Table 7.** **Antibodies used in this study** | | | |
| --- | --- | --- | --- |
| Antibody | Dilution ratio | Manufacturer | Catalog |
| TGFβ2 | 1:1000 | Proteintechm | 19999-1-AP |
| TGFβ2 (IHC) | 1:200 | Bio-TechneBio-Techne | MAB612-100 |
| NDRG1 (IF) | 1:200 | Abcam | ab124689 |
| NDRG1 (IHC) | 1:1000 | Abcam | ab37897 |
| NDRG1 | 1:10000 | Abcam | ab37897 |
| IgG (IF) | 1:800 | Thermo Fisher Scientific | A-21428 |
| E-cadherin | 1:1000 | Abcam | ab314063 |
| N-cadherin | 1:5000 | Abcam | ab76011 |
| Smad2/3 | 1:1000 | Cell Signaling Technology | #8685 |
| pSmad2-S465/S467 | 1:5000 | ABclonal | AP1342 |
| pSmad3-S423/S425 | 1:2000 | ABclonal | AP0727 |
| pSmad3 (ChIP) | 1:100 | Thermo Fisher Scientific | # MA5-14936 |
| GAPDH | 1:5000 | Proteintech | HRP-60004 |
| KIAA1199 | 1:1000 | Proteintechm | 21129-1-AP |

| **Supplementary Table 8. siRNA used in this study** | | |
| --- | --- | --- |
| Gene | Forward primer  sense（5'-3'） | Reverse primer  antisense（5'-3'） |
| NDRG1 | AACCUGCUACAACCCCCUCTT | GAGGGGGUUGUAGCAGGUUTT |
| Smad2 | GAAUUGAGCCACAGAGUAA | UUACUCUGUGGCUCAAUUC |
| Smad2 | GGAUUGAACUUCAUCUGAA | UUCAGAUGAAGUUCAAUCC |
| Smad3 | GCUUGGUGAAGAAGCUCAA | UUGAGCUUCUUCACCAAGC |
| Smad3 | CCAGAGCAAUAUUCCAGAA | UUCUGGAAUAUUGCUCUGG |
| siNC | UUCUCCGAACGUGUCACGU | ACGUGACACGUUCGGAGAA |


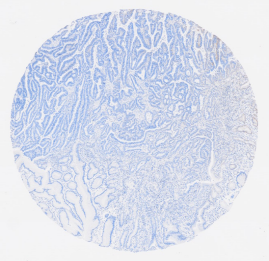

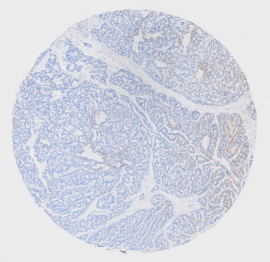

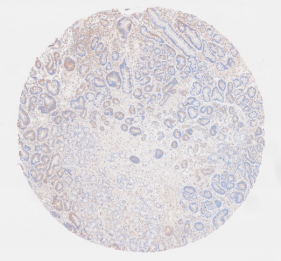

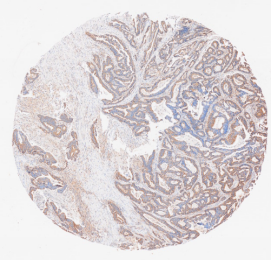

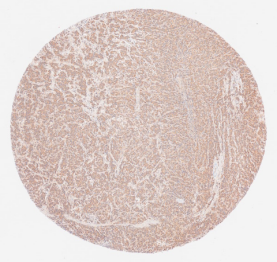

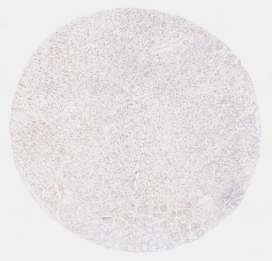

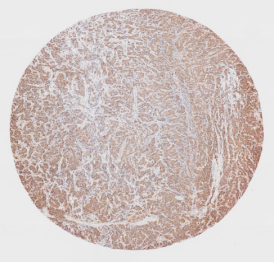


0.0527

Mean value of DAB

0.0976

0.0702

0.031

0.1599

0.1399

0.0049

0.1768


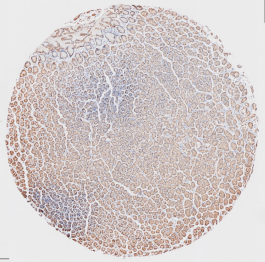


Mean value of DAB

**Supplementary Table 9. Partial staining score presentation for DAB score by QuPath**
